# Impact of climate change on the distribution of *Isaria cicadae* *Miquel* in China: predictions based on the MaxEnt model

**DOI:** 10.3389/fmicb.2025.1509882

**Published:** 2025-02-07

**Authors:** Zhipeng He, Habib Ali, Junhao Wu, Zhiqian Liu, Xinju Wei, Zhihang Zhuo

**Affiliations:** ^1^College of Life Science, China West Normal University, Nanchong, China; ^2^Department of Agricultural Engineering, Khwaja Fareed University of Engineering and Information Technology, Rahim Yar Khan, Pakistan

**Keywords:** *Isaria cicadae*, MaxEnt, climate change, potential distribution, environmental

## Abstract

**Introduction:**

*Isaria cicadae*, a historically valued edible and medicinal fungus in China, has been experiencing a critical decline in abundance due to ecological degradation and overexploitation. Understanding its potential distribution is essential for promoting sustainable harvesting practices.

**Methods:**

This study utilizes the MaxEnt model, combined with known distribution records and 22 environmental variables, to predict the potential distribution of *I. cicadae* under three representative emission scenarios (CMIP6: SSP1-2.6, SSP2-4.5, and SSP5-8.5) for the 2050s and 2070s.

**Results:**

The analysis identifies seven key environmental variables influencing the habitat suitability of *I. cicadae*: the mean temperature of the driest quarter (bio09), the mean temperature of the wettest quarter (bio08), precipitation in the wettest month (bio16), the mean diurnal range (bio02), isothermality (bio03), elevation, and slope. Currently, *I. cicadae* is mainly found in the provinces of Yunnan, Sichuan, Hunan, Hubei, Guizhou, Jiangxi, Guangxi, Fujian, Anhui, and Zhejiang, with Yunnan and Sichuan having the largest areas of high suitability at 25.79 × 10^4^ km^2^ and 21.36 × 10^4^ km^2^, respectively.

**Discussion:**

Jiangxi, Hunan, Yunnan, Guizhou, Fujian, and the Guangxi Zhuang Autonomous Region are identified as primary regions of high suitability. This study aims to further elucidate the impact of the environment on the distribution of *I. cicadae* from a geographical perspective and provide theoretical insights for the future cultivation and conservation strategies of this species.

## 1 Introduction

*Isaria cicad*ae *Miquel* is a fungus of the genus *Isaria*, belonging to the family Cordycipitaceae and the order Hypocreales (Shiwei and Hongkai, 2023; Zhaoying, 2020). This species originates from the invasion of cicada larvae by fungi, where the fungal hyphae rapidly proliferate, ultimately leading to the death of the larva. The seven main hosts of this species in China are *Platypleura kaempferi*, *Platylomia pieli*, *Cicada flammata*, *Mogannia conica conica*, *Oncotympana ella*, *Cicadatra shaluensis*, and *Hyalessa ronsnana* (Jiao, 2021). The resulting mycelial complex, which matures during the late spring and early summer (Liu et al., 2008). The species predominantly inhabits bamboo forests, broadleaf forests at altitudes below 2,500 meters, or mixed coniferous-broadleaf forests dominated by species such as *Cyclobalanopsis glauca*, *Castanea henryi*, *Pinus yunnanensis*, and *Abies* spp. (Wang et al., 2006; Zhang et al., 2022). *I. cicadae* is a highly regarded *Cordyceps* species, similar to the well-known *Ophiocordyceps sinensis*. It is rich in bioactive compounds such as nucleosides, ergosterol, cordycepic acid, and polysaccharides, which have considerable potential for both culinary and medicinal uses (Zhu et al., 2024). Research indicates that *I. cicadae* has health benefits similar to those of *O. sinensis* and holds significant market value (Bian et al., 2017). However, global climate change and human activities have severely degraded its habitat, leading to a sharp decline in its population and a mismatch between supply and demand. The International Union for Conservation of Nature (IUCN) has classified this species as vulnerable on its Red List (Li et al., 2020). Therefore, it is significant to identify and predict regions suitable for the cultivation and conservation of *I. cicadae*.

Species distribution models (SDMs) are empirical tools used to quantify the ecological niches of species within their environments. SDMs are created by integrating occurrence data of species with corresponding environmental variables, identifying associations between them, and using these relationships to estimate the species’ distribution across the study area (Zhonglin et al., 2015). By combining these occurrence data with environmental variables from sampling sites, SDMs model the species-environment relationships to predict potential distribution patterns (Deng et al., 2022). Commonly used niche models include CLIMEX, BIOCLIM, GARP, DOMAIN, and MaxEnt (Wei et al., 2024). The MaxEnt model is particularly notable for its advantages, such as its effectiveness with small sample sizes, high predictive accuracy, rapid computation, and user-friendliness. In addition, it can use the Jackknife method to conduct significance testing and evaluation on individual environmental variables. The MaxEnt model has been widely applied both domestically and internationally across various fields (Brambilla et al., 2024; Zhao et al., 2024). In particular, MaxEnt is extensively used in disciplines such as ecology (Cao et al., 2023), conservation biology (Lin et al., 2024), and geography (Wang C. et al., 2023; Wang Z. et al., 2023). Its applications include assessing the potential impacts of climate change (Guo et al., 2017; Guo et al., 2019), predicting the potential distributions of endangered species (Ab Lah et al., 2021; Lin et al., 2022), evaluating the spread of invasive species (Bowen and Stevens, 2020; Tu et al., 2021), forecasting changes in ecological habitat ranges (Wang et al., 2024), and investigating biomass reserves (Rodríguez-Veiga et al., 2016).

Research on the distribution of *I. cicadae* is currently limited. Existing studies primarily concentrate on its edibility (Jia et al., 2023), chemical composition (Mei et al., 2023), metabolomic profiling (Tang et al., 2023), and the pharmacological potential of its key compounds (Li Z. et al., 2024; Olatunji et al., 2016). Investigations into suitable habitats for *I. cicadae* are notably scarce. Therefore, this study provides valuable scientific evidence for exploring the potential distribution of *I. cicadae*.

Using known distribution data for *I. cicadae* and WorldClim environmental variables, this study employed ArcGIS 10.8 and MaxEnt 3.4.4 to analyze the environmental adaptability of *I. cicadae*. This study predicts both the current and future potential geographical distribution of *I. cicadae* and examines the trends in centroid movement affecting its distribution. Additionally, this study identifies key environmental factors influencing the distribution of *I. cicadae* and assesses their impact on the fungus, providing theoretical insights for its conservation.

## 2 Materials and methods

### 2.1 Species distribution data

**Figure 1:**
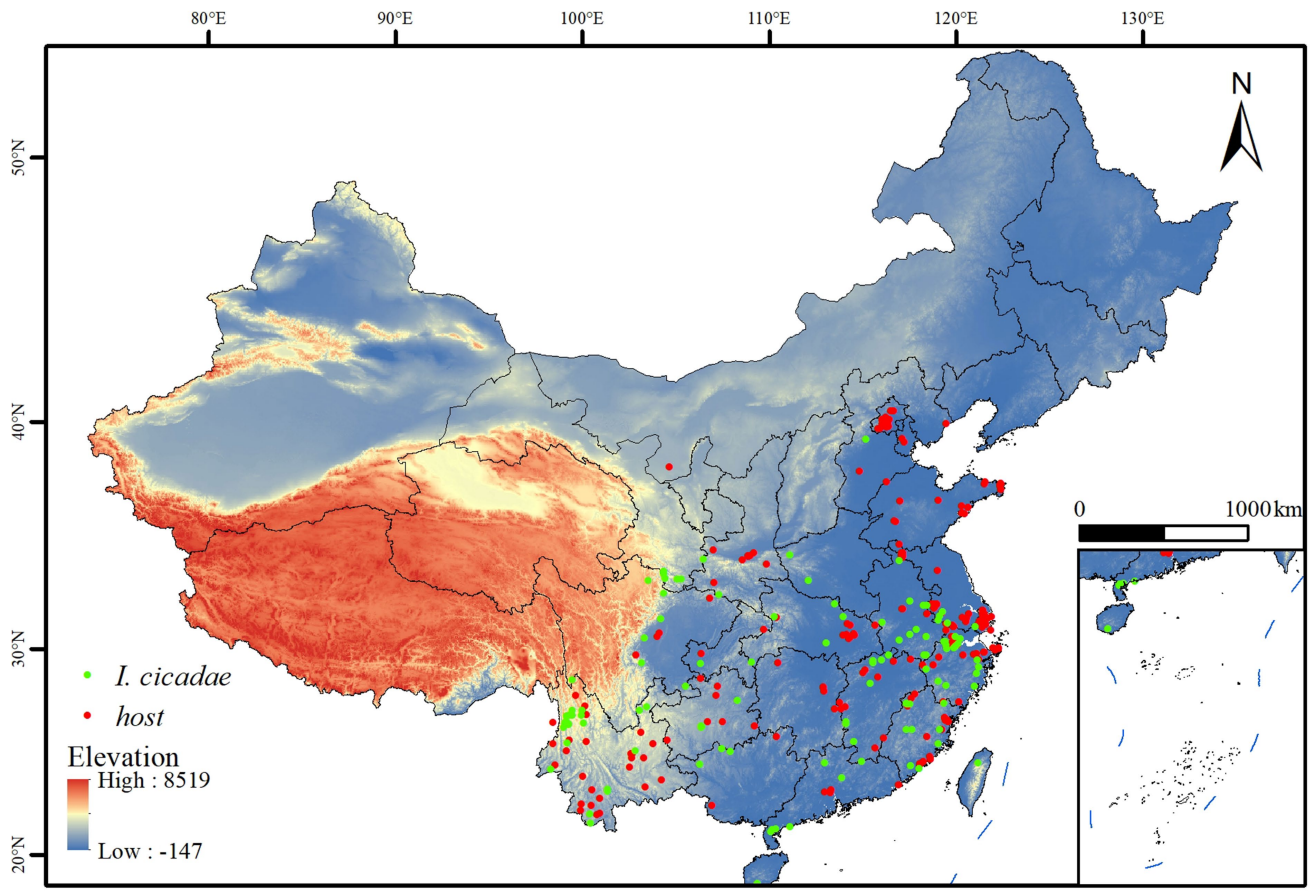
Geographical distribution points of *I. cicadae* in China.

This study primarily obtained species distribution data for *I. cicadae* from the Global Biodiversity Information Facility (GBIF),^1^ including 194 occurrence records for *I. cicadae* and 634 host distribution data points. The precise geographic coordinates of the species distribution points were determined using Google Earth.^2^ The collected distribution points for *I. cicadae* were imported into ArcGIS 10.8. Buffer analysis was employed to filter these distribution points, reducing overfitting caused by spatial overlap or proximity. The filtered records were saved in CSV format, resulting in a total of 161 *I. cicadae* occurrence records and 247 host occurrence records, as shown in Figure 1.

### 2.2 Environmental variables and data processing

This work downloaded 19 climate variables from the WorldClim database.^3^ To predict the potential distribution of *I. cicadae* under future climate conditions, the study employed three scenarios from the Beijing Climate Center’s (BCC) advanced climate model BCC-CSM2-MR: SSP1-2.6 (low emission pathway), SSP3-7.0 (medium emission pathway), and SSP5-8.5 (high emission pathway). To address high autocorrelation among environmental variables and to reduce overfitting while improving model accuracy, Pearson correlation coefficients (*r*) were used to identify multicollinearity among the environmental variables (Table 1). By excluding variables with correlation coefficients greater than 0.8, the impact of multicollinearity on overfitting was minimized (Fu et al., 2024). This process ultimately determined the key environmental variables required for modeling.

**Table 1:** Presents Pearson correlation coefficients among the environmental factors.

	slope	bio2	bio3	bio8	bio16	bio19
bio2	0.0930					
bio3	0.0748	0.4152				
bio8	0.1755	0.5345	0.5265			
bio16	0.1625	0.0872	0.6396	0.4919		
bio19	0.0836	−0.0984	0.4786	0.2348	0.6506	
elev	0.0775	−0.1737	−0.2317	−0.7654	−0.3474	−0.2275

### 2.3 Model construction and assessment

Distribution data for *I. cicadae* with various environmental variables to assess their impact on the species distribution. Using MaxEnt version 3.4.4, the potential distribution range of the species was modeled. To minimize the uncertainty introduced by random data selection, 75% of the species occurrence data was randomly designated as training samples, with the remaining 25% reserved for testing. The training process was repeated 10 times, keeping all other parameters at their default settings. This study employed the jackknife technique to determine the contribution rates of key environmental variables and utilized response curves to analyze the relationship between *I. cicadae* distribution and climatic variables.

The MaxEnt model quantifies species suitability on a scale from 0 to 1, with values closer to 1 indicating a higher probability of the species’ presence under specific environmental conditions (Li R. et al., 2024). Following the Intergovernmental Panel on Climate Change (IPCC) reporting methodology and considering the habitat suitability for *I. cicadae*, regions with a suitability score below 0.1 were classified as unsuitable. Areas with scores between 0.1 and 0.3 were designated as low suitability, those between 0.3 and 0.5 as moderate suitability, and habitats with scores of 0.5 or higher were considered highly suitable (Iturbide et al., 2020).

The model performance is evaluated using receiver operating characteristic (ROC) curve, which is essential for assessing the accuracy of its predictions. A key metric in this evaluation is the area under the ROC curve (AUC), which measures the effectiveness of the MaxEnt model (Wang C. et al., 2023; Wang Z. et al., 2023). The AUC ranges from 0.5 to 1, with values closer to 1 indicating higher model precision (Walden-Schreiner et al., 2017). Specifically, an AUC between 0.5 and 0.6 reflects substandard performance, 0.6 to 0.7 indicates poor performance, 0.8 to 0.9 signifies excellent performance, and values above 0.9 denote high performance (Bai et al., 2024).

## 3 Results

### 3.1 Model performance and key environmental variables

**Figure 2:**
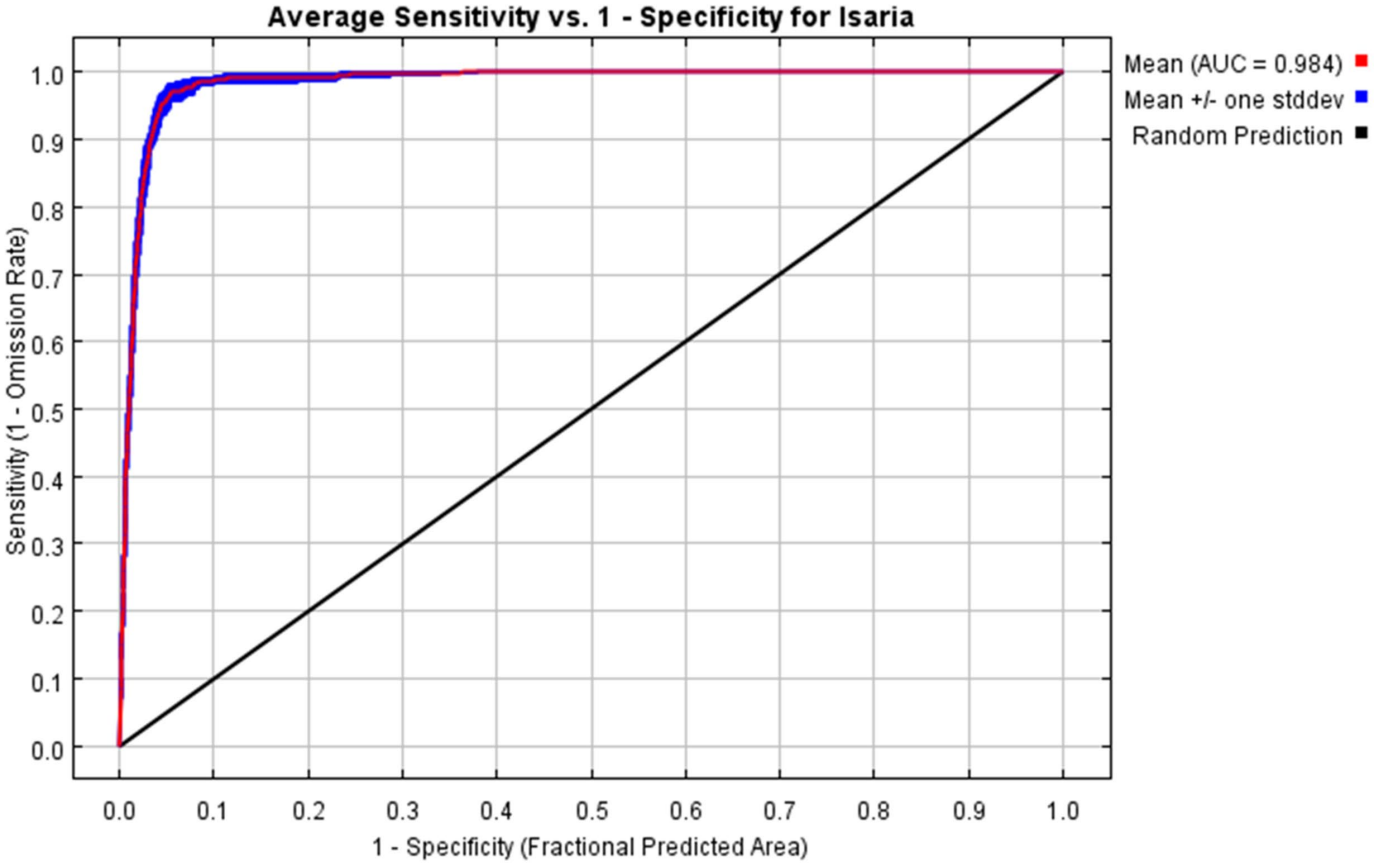
Receiver operating characteristic curve and AUC result of MaxEnt modeling.

The area under the curve (AUC) is a metric that reflects the accuracy of MaxEnt model simulations. In this study, the MaxEnt model achieved an average AUC of 0.984 ± 0.002, as shown in Figure 2. This high level of accuracy underscores the model’s reliability and supports its use as a foundation for the current investigation.

Using Pearson correlation coefficients, seven key environmental variables were identified and incorporated into the species distribution model. Jackknife analysis revealed the contribution of each variable as follows: bio16 (50.7%), bio19 (20.0%), slope (7.9%), bio03 (7.1%), bio08 (6.2%), bio02 (6.1%), and elevation (1.9%), with a cumulative contribution of 99.9%. In terms of importance, the rankings were: bio09 (42.9%), bio08 (25.7%), bio16 (20.6%), bio02 (5.5%), bio03 (2.6%), elevation (1.5%), and slope (1.2%), with a total importance score of 100% (Table 2). These results indicate that the selected environmental variables effectively simulate the potential distribution of *I. cicadae*.

**Table 2:** The percent contribution and permutation importance of climatic variables in the MaxEnt modeling of *I. cicadae*.

Variable	Percent contribution	Permutation importance
bio16	50.7	20.6
bio09	20	42.9
slope	7.9	1.2
bio03	7.1	2.6
bio08	6.2	25.7
bio02	6.1	5.5
elev	1.9	1.5

### 3.2 The potential distribution of *Isaria cicadae* in the current period

**Figure 3:**
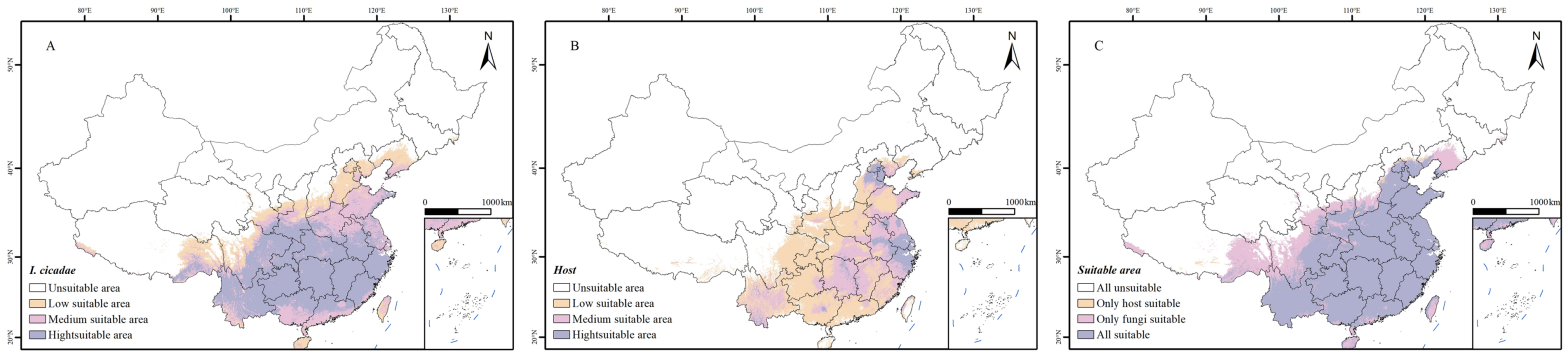
Under the current situation, the potential suitable distribution area of *I. cicadae* and host. **(A)** The potential suitable distribution area of *I. cicadae*. **(B)** The potential suitable distribution area of host. **(C)** Suitable area overlay map.

Employing an MaxEnt model, a predictive map of the most suitable habitats for *I. cicadae* was generated (Figure 3A). The findings suggest that under the current climate scenario, the species’ suitable habitats are widely distributed across most of China, excluding the provinces of Jilin, Heilongjiang, Inner Mongolia, Qinghai, Ningxia, and Xinjiang. The total area of suitable habitats covers approximately 341.54 × 10^4^ km^2^, equivalent to 35.53% of China’s land area. High-suitability regions are predominantly situated in Yunnan, Sichuan, Hunan, Hubei, Guizhou, Jiangxi, Guangxi, Fujian, Anhui, and Zhejiang, covering an area of roughly 194.34 × 10^4^ km^2^, which represents 20.22% of China’s total landmass. Currently, Yunnan Province boasts the largest area of high suitability, encompassing 25.79 × 10^4^ km^2^, followed by Sichuan Province with 21.36 × 10^4^ km^2^ and Hunan Province with 19.13 × 10^4^ km^2^. These regions account for 13.27, 10.99, and 9.85% of the national high-suitability habitat area, respectively. Notably, the high-suitability area in Hunan constitutes 98.90% of the province’s total land area, while in Jiangxi and Guizhou, these proportions are even more significant, reaching 99.23 and 99.27%, respectively. Additionally, in Zhejiang, Hubei, and Fujian, the high-suitability areas surpass 80% of their respective provincial and masses (see Table 3).

The potential distribution areas of the primary hosts for *I. cicadae* under the current climate scenario were also predicted (Figure 3B). High-suitability regions for the hosts span an area of 24.884 × 10^4^ km^2^, primarily located at the junction of Zhejiang, Jiangsu, and Anhui provinces. Furthermore, when the suitable areas from the *I. cicadae* potential distribution map were overlaid with those of the host’s potential distribution (Figure 3C), it was found that the shared suitable region for both *I. cicadae* and the hosts spans 267.089 × 10^4^ km^2^, representing 78.41% of the suitable area for *I. cicadae* and 99.13% of the suitable area for the hosts.

**Table 3:** *I. cicadae* top 10 high-suitability regions for habitat.

Province	High suitable area (10^4^ km^2^)	Total (10^4^ km^2^)	Percentage of highly suitable areas in the province (%)	Percentage of highly suitable areas in China (%)
Yunnan	25.79	34.26	75.29%	13.27%
Sichuan	21.36	45.74	46.70%	10.99%
Hunan	19.13	19.35	98.90%	9.85%
Hubei	16.37	17.57	93.15%	8.42%
Guizhou	15.87	15.99	99.27%	8.17%
Jiangxi	15.13	15.25	99.23%	7.78%
Guangxi	12.38	20.89	59.28%	6.37%
Fujian	9.68	10.94	88.45%	4.98%
Anhui	9.51	13.37	71.13%	4.89%
Zhejiang	8.81	9.36	94.14%	4.53%
China	194.34			20.22%

### 3.3 The potential distribution of *Isaria cicadae* in the future period

**Figure 4:**
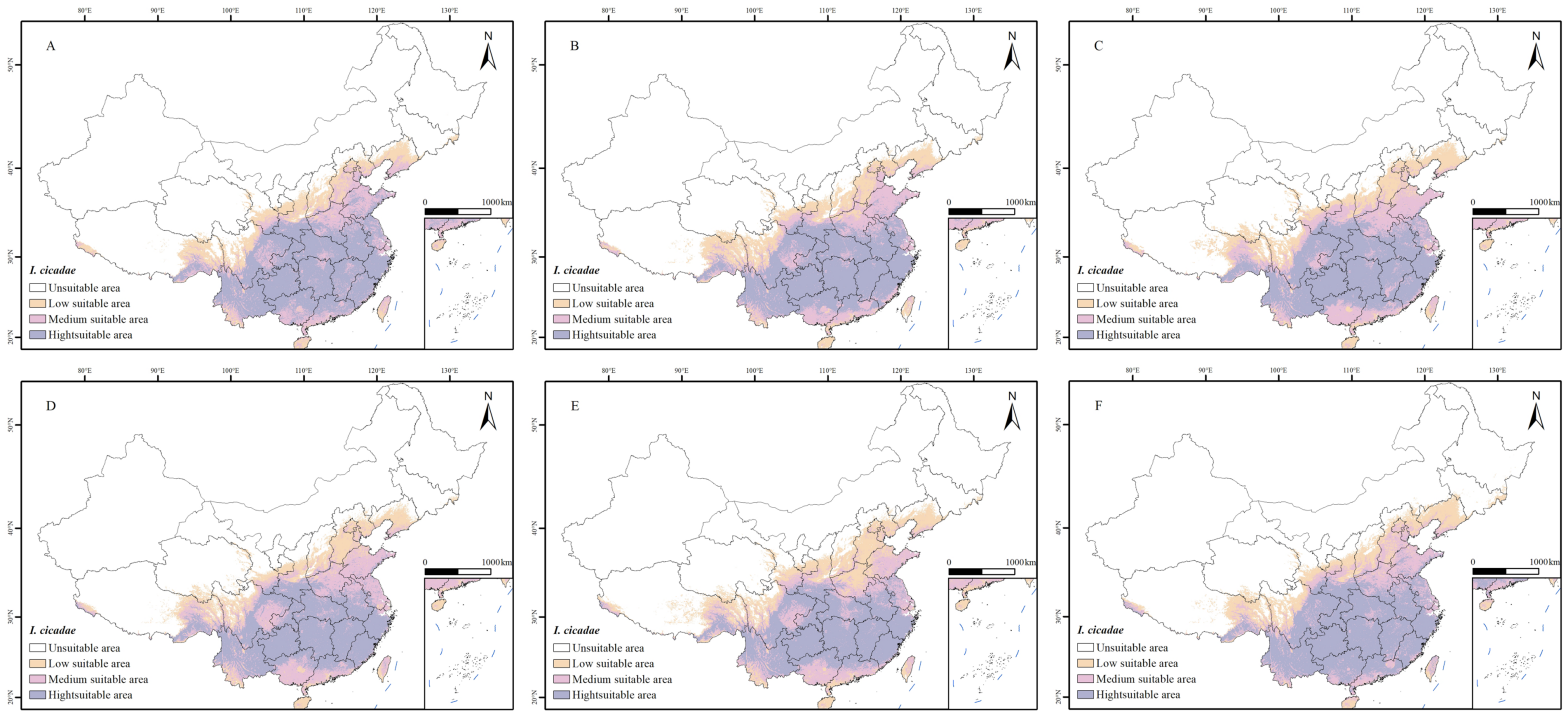
A predictive map of potentially suitable areas for *I. cicadae* in China under different climate change scenarios. **(A)** SSP1-2.62050s. **(B)** SSP3-7.02050s. **(C)** SSP5-8.52050s. **(D)** SSP1-2.62070s. **(E)** SSP3-7.02070s. **(F)** SSP5-8.52070s. (Purple: high suitability zone; pink: moderate suitability zone; yellow: low suitability zone; white: unsuitable zone).

Under the climate change scenarios of SSP1-2.6, SSP3-7.0, and SSP5-8.5, the suitable distribution ranges for *I. cicadae* in the periods 2041–2060 and 2061–2080 are illustrated in Figure 4. Exceptions for Xinjiang, Qinghai, Ningxia, Inner Mongolia, Heilongjiang, and Jilin Provinces, suitable habitats for *I. cicadae* are found across the rest of China. High-suitability zones are primarily located in Chongqing, Zhejiang, Jiangxi, Hunan, Yunnan, Guizhou, Sichuan, Fujian, Guangdong, and Guangxi. Additionally, there are scattered areas of high suitability in Gansu, Shaanxi, Henan, and Hebei. The projected distribution regions for the 2050s and 2070s show minimal changes compared to the current distribution. In the 2050s, high-suitability areas only decreased under the SSP3-7.0 and SSP5-8.5 scenarios, while other suitability categories expanded. By the 2070s, high-suitability areas decreased under the SSP1-2.6 and SSP3-7.0 scenarios, while moderate-suitability areas shrank under the SSP5-8.5 scenario, with other categories continuing to expand (Table 4).

**Table 4:** Predicts suitable areas for *I. cicadae* under the current and future climatic conditions.

Decade scenarios	Predicted area (10^4^ km^2^)			Comparison with current distribution (%)		
	Low suitable	Medium suitable	High suitable	Low suitable	Medium suitable	High suitable
Current	61.72	85.49	194.34			
41-60ssp126	66.63	87.00	195.60	7.96%	1.77%	0.65%
41-60ssp370	77.78	89.28	189.38	26.02%	4.44%	−2.55%
41-60ssp585	75.95	101.35	187.44	23.06%	18.55%	−3.55%
61-80ssp126	74.72	105.73	176.25	21.07%	23.67%	−9.31%
61-80ssp370	93.46	95.72	171.32	51.43%	11.97%	−11.84%
61-80ssp585	74.92	84.86	206.04	21.39%	−0.73%	6.02%

The predictions suggest that the suitable habitat area for *I. cicadae* will generally trend upward during the 2050s. However, under the SSP3-7.0 and SSP5-8.5 scenarios, the high suitability region for *I. cicadae* is expected to decrease by 2.55 and 3.55%, respectively. In contrast, the other suitable habitat areas for *I. cicadae* are projected to increased.

The findings suggest that by the 2070s, the suitable habitat for *I. cicadae* is projected to expand overall. However, this expansion is not uniform: significant growth is observed only under the SSP5-8.5 scenario in high-suited areas, with an increase of approximately 6.02%. In contrast, a decrease in suitable habitat area is evident only within the SSP5-8.5 scenario for mid-suited areas, with a reduction of about 0.73%. Across all three scenarios, low-suited areas exhibit an increase in habitat extent.

### 3.4 The environmental variables influencing the geographical distribution of *Isaria cicadae*

**Figure 5:**
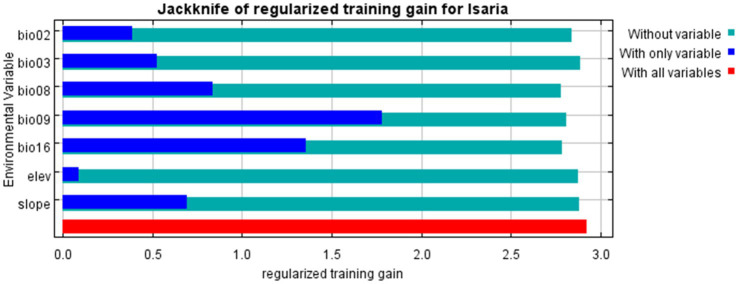
The jackknife test assesses the importance of environmental variables for *I. cicadae*.

This study evaluated three scenarios-“only variables,” “no variables” and “all variables”-to assess the influence of environmental factors on the distribution of *I. cicadae* using MaxEnt software and the jackknife method. The analysis demonstrated the varying impacts of seven environmental variables on the species’ distribution (Figure 5). The results exhibit that when using only a single environmental variable, bio09 and bio16 have the most significant effect on the model’s performance. This finding highlights that these two factors play a critical role in shaping the distribution of *I. cicadae*.

**Figure 6:**
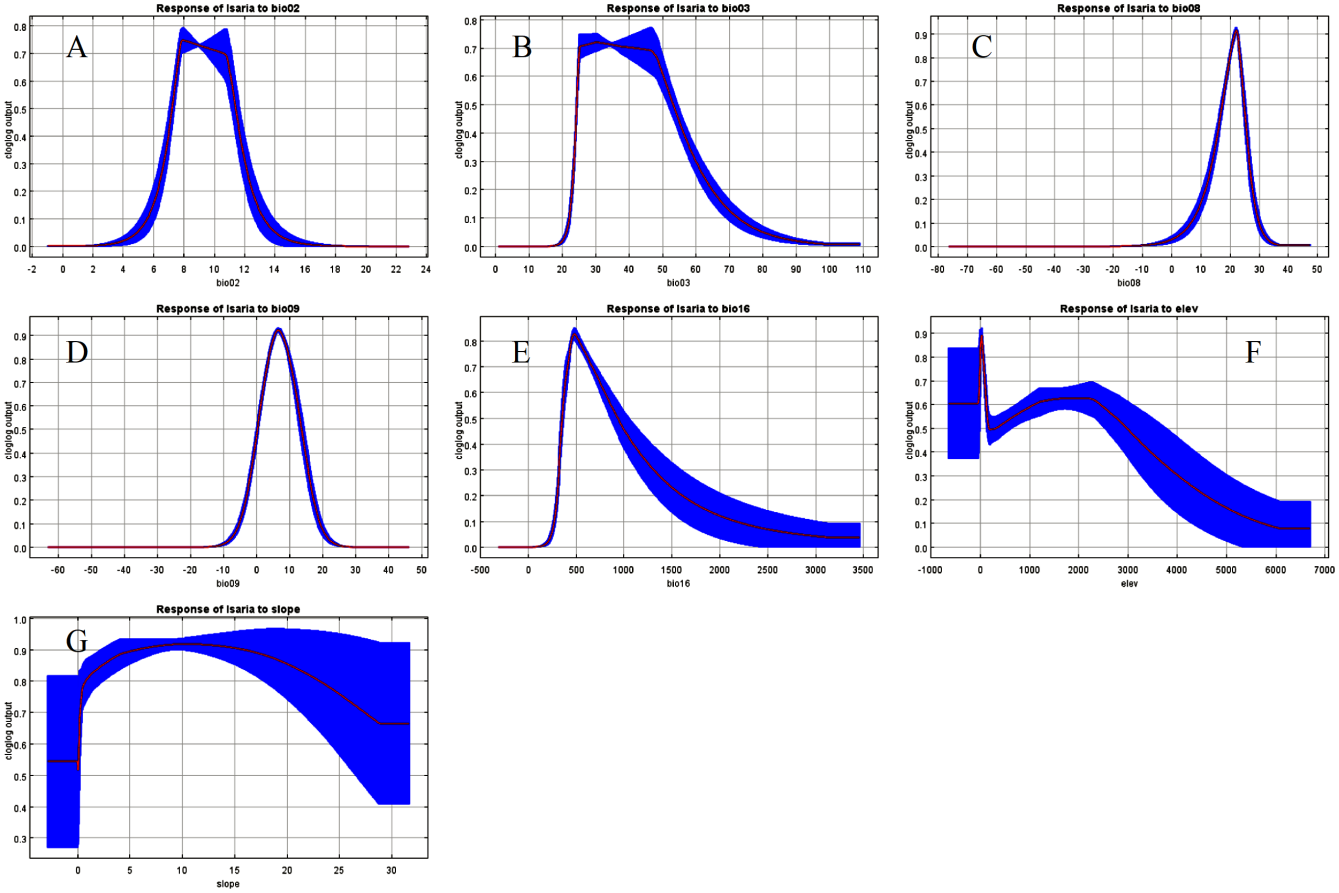
The probability of the presence of *I. cicadae* and the response curve of key environmental variables. **(A)** bio02. **(B)** bio03. **(C)** bio08. **(D)** bio09. **(E)** bio16. **(F)** elev. **(G)** slope. The red curve represents the average of 10 replicates; blue margins represent the standard deviation.

Based on the response curves of the *I. cicadae* probability distribution shown in Figure 6, with a threshold of 0.5, values above 0.5 are considered suitable. The suitable ranges for the seven environmental variables—bio02, bio03, bio08, bio09, bio16, elevation, and slope—are as follows: bio02: 7.18 to 11.39°C, bio03: 24.45 to 53.28°C, bio08: 16.05 to 25.62°C, bio09: 0.23 to 13.70°C, bio16: 352.78 to 936.53 mm, elevation: less than 2,990 m, and slope: greater than 1.71 (Table 5).

**Table 5:** The optimal range of environmental variables corresponds to the potential distribution of *I. cicadae*.

Environmental variables	Suitable range	Optimum value
bio02/°C	7.18–11.39	7.89
bio03/°C	24.45–53.28	29.88
bio08/°C	16.05–25.62	22.37
bio09/°C	0.23–13.70	6.62
bio16/mm	352.78–936.53	484.88
elev	<2,990	24.8
slope	>1.71	14.49

### 3.5 The centroid variation in the potential distribution of *Isaria cicadae*

**Figure 7:**
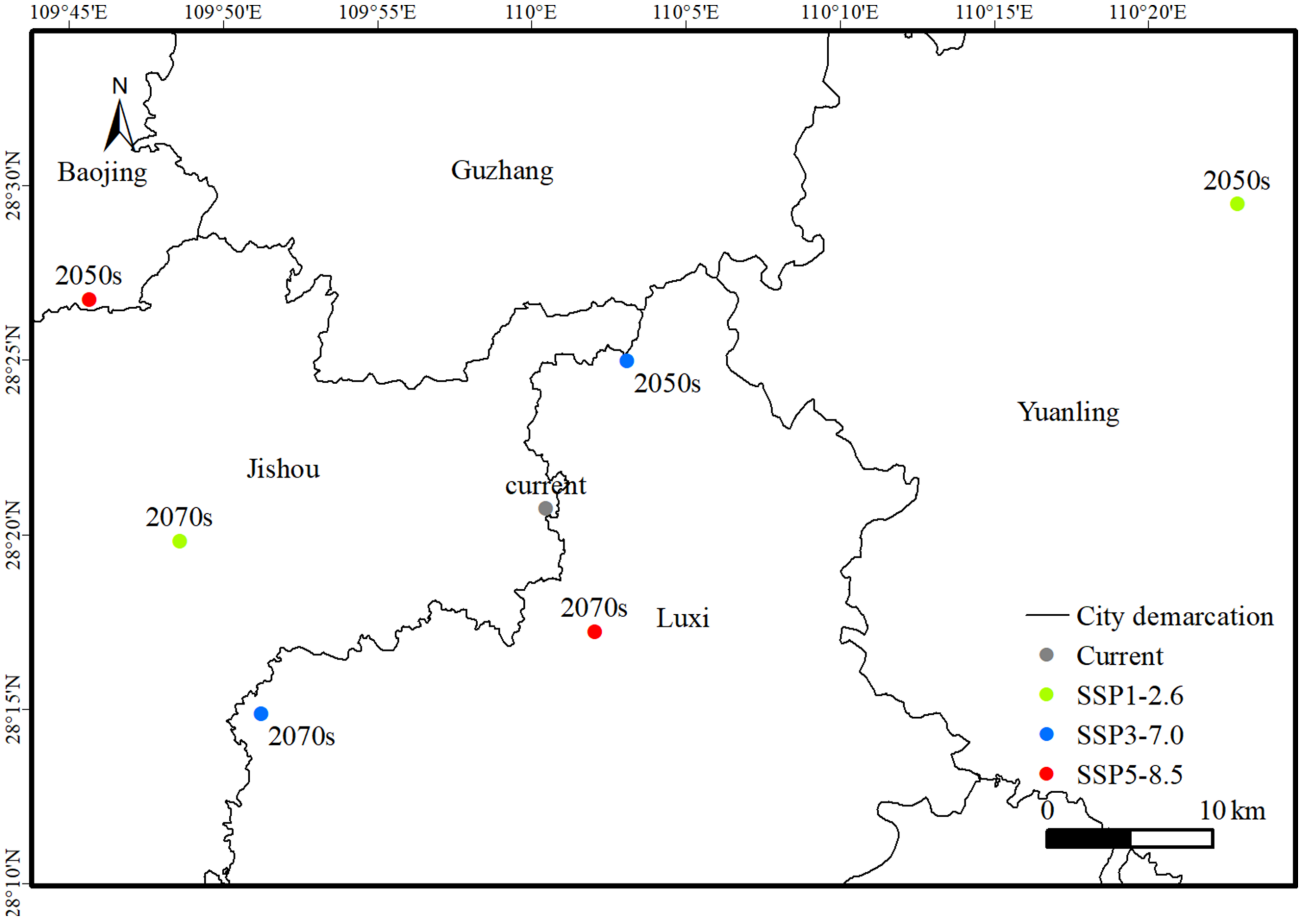
The change in the centroid of the potential distribution area of *I. cicadae* in China.

Figure 7 illustrates the high suitability centroids of *I. cicadae* under different climate scenarios. Currently, the centroid of the high suitability zone is located in Jishou City, Hunan Province. In the SSP1-2.6 scenario, by the 2050s, the centroid is projected to shift northeast by 43.14 km to Yuanling County. By the 2070s, it is expected to move southwest by 58.89 km, returning to Jishou City. In the SSP370 scenario, by the 2050s, the centroid is anticipated to move northeast by 8.95 km into Luxi County. By the 2070s, it is expected to shift southwest by 26.99 km, remaining within Luxi County. Under the SSP585 scenario, by the 2050s, the centroid is projected to shift northwest by 26.63 km into Baojing County, and by the 2070s, to move southeast by 32.11 km into Luxi County. Among the three carbon emission scenarios, centroid displacement is relatively minor under SSP3-7.0 and SSP5-8.5 from 2050 to 2070, while it is more pronounced under SSP1-2.6.

In summary, except for the 2050s under the SSP5-8.5 and SSP1-2.6 scenarios, the potential high suitability areas for *I. cicadae* are predominantly located within Jishou and Luxi Counties (Table 6). This suggests that the distribution of suitable habitats for *I. cicadae* is influenced by the selected climate scenario, with notable shifts anticipated under specific scenarios.

**Table 6:** Under climate change scenarios, the centroid displacement trajectory of *I. cicadae* habitats.

Scene	Period	Direction	Displacement/km	Location
ssp126	Contemporary to 2050s	Northeast	40.14	Yuanling county
	2050s to 2090s	Southwest	58.89	Jishou city
ssp370	Contemporary to 2050s	Northeast	8.95	Luxi county
	2050s to 2090s	Southwest	26.99	Luxi county
ssp585	Contemporary to 2050s	Northwest	26.63	Baojing county
	2050s to 2090s	Southeast	32.11	Luxi county

## 4 Discussion

The MaxEnt model is widely used in species distribution modeling. In this study, MaxEnt version 3.4.4 was used in combination with 19 climatic variables and 3 topographic factors to model and predict species distribution under current and potential future climatic conditions. ArcGIS version 10.8 was then integrated to analyze the spatial distribution of *I. cicadae*. While some studies, such as those by Kumar et al. (2014), focus exclusively on climatic variables and omit topographic factors, this study adopts a combined approach that integrates both climatic and topographic variables for a more comprehensive analysis. The findings indicate that *I. cicadae* is less influenced by topographic variables and is more significantly affected by climatic factors. MaxEnt model predictions reveal a concentrated distribution of suitable habitats for *I. cicadae*, predominantly in Southwestern, Southern, Central, and Eastern China, with highly suitable habitats concentrated in provinces surrounding the Yangtze River basin. In future climate scenarios, the range of suitable habitats for *I. cicadae* shows slight expansion, with an increase in areas classified as low and moderate suitability, while the extent of high suitability decreases. A trend toward habitat expansion at higher latitudes is observed, while some low-latitude regions experience a reduction in suitability or even a loss of suitable habitats. Currently, Yunnan Province hosts the largest high-suitability area, but future projections suggest a decrease in the high-suitability area within Yunnan, accompanied by an expansion of the moderate-suitability region. Analysis of Figure 4 and Table 4 indicates that, over time, the high-suitability area for *I. cicadae* is increasing and extending northward, remaining concentrated in Yunnan, Sichuan, Jiangxi, Hubei, Hunan, Chongqing, and Guizhou provinces.

The MaxEnt model, utilizing a combination of climate, topographic, and *I. cicadae* distribution data, was employed to assess habitat suitability. The model’s AUC value is 0.984, which provides robust support for the study. This study employed Pearson correlation coefficients to select seven pivotal environmental variables (bio02, bio03, bio08, bio09, bio16, elevation, and slope) as significant influencers on the distribution of *I. cicadae*. Jiaojiao et al. (2018) demonstrated that the suitable habitat of *I. cicadae* is influenced by factors such as temperature and light. In parallel, Zhu et al. (2017) investigated the relationship between Cordycipitaceae biomass and climatic conditions, revealing that the yield of Cordycipitaceae is, to some extent, influenced by precipitation levels. The results of this study further support this conclusion. In their phylogenetic study of parasitic fungi, Gorczak and Trigos-Peral (2021) observed that infection typically occurs during the warm, humid season when temperatures are favorable. Infection happens when older larvae active in shallow soil layers come into contact with soil contaminated with fungal spores, leading to the development of mature forms. Additionally, the jackknife test was utilized to assess the significance of these seven environmental variables in influencing the distribution of *I. cicadae*. In ranking the significance of the seven key environmental variables, the mean temperature during the wettest quarter (bio08), the rainfall during the wettest season (bio16), and the mean temperature of the driest quarter (bio09) occupied the top three positions, with their importance increasing sequentially. This alignment with the findings of the three preceding researchers suggests the reliability of our results, indirectly validating their credibility.

Based on the overlap analysis of the potential distributions of the hosts and *I. cicadae*, the potential distribution of *I. cicadae* largely overlaps with that of its primary hosts, with the hosts’ suitable areas covering 78.41% of *I. cicadae’s* suitable areas, and vice versa, 99.13%. This provides strong validation of the model’s predictive accuracy. The smaller suitable area for the hosts compared to *I. cicadae* is attributed to the presence of other hosts (Xu et al., 2025). These results emphasize the potential impact of climate change on species distribution, particularly the shared adaptability of hosts and parasitic species, which could lead to species expansion or contraction in specific regions in the future (Wei et al., 2023).

Under the three classic climate scenarios of SSP126, SSP370, and SSP585, the area of suitable habitats is projected to increase by the 2050s and 2070s. The predictive results indicate that high-suitability areas will continue to be concentrated in regions such as Guizhou, Hunan, and Jiangxi. However, there is a decrease in the size of these high-suitability areas, a trend that is particularly pronounced in the 2050s under the SSP126 scenario. Comparing the predicted results across different climate scenarios, it is observed that under the SSP126 scenario, the increase in suitable areas is less pronounced in the 2050s than under the other two scenarios. From 2050 to 2070, the increase in suitable habitat area for *I. cicadae* is more pronounced than in other scenarios, suggesting a potential enhancement in the species’ adaptability. Currently, the center of mass for the species is situated in Jishou City, Hunan Province (110°0′27″ E, 28°20′45″ N). Projections suggest that, except for the 2050s under the SSP585 and SSP126 scenarios, the center of mass for *I. cicadae* will remain within the coordinates of 109°48′37″ E to 110°3′6″ E and 28°14′52″ N to 28°24′59″ N, covering the areas of Jishou and Luxi counties. The negligible displacement of the center of mass suggests that there is no significant directional change in habitat suitability for the species.

When employing species distribution models for predicting potential distributions, it is common practice to consider the complex interplay of multiple biotic factors and environmental variables that influence species distribution (Maurya et al., 2023). The MaxEnt model used in this study integrates data on the known distribution of the target species with environmental variables to estimate the probability of species distribution. While MaxEnt demonstrates strong predictive capabilities, it has notable limitations, including the restriction to temperature, precipitation, and topography as environmental variables, which may overlook other potential determinants. Despite its widespread use for predicting species distributions, MaxEnt has inherent limitations (Xu et al., 2019). The response curve illustrates the impact of a single environmental factor without considering the complex interactions among multiple variables. Given the practical limitations of incorporating all environmental factors comprehensively in model development and analysis, it is more appropriate to use this model as a foundational niche model to accurately capture ecological principles (Lin et al., 2022; Weiyao et al., 2019). As a fungal species, the survival of *I. cicadae* is influenced not only by its biological traits but also by environmental factors such as host distribution, light conditions, and human activities (He et al., 2024; Shi et al., 2015). However, this study has certain limitations, particularly regarding the potential impact of anthropogenic factors and soil variables on species distribution. Future research should incorporate additional factors, particularly biotic factors such as host types and abiotic factors such as human activities, to provide a more comprehensive analysis and improve the accuracy of the model’s predictions.

## 5 Conclusion

In this study, distribution data for *I. cicadae* were integrated with three topographic and 19 bioclimatic variables using the MaxEnt model. This integration successfully modeled the potential geographic distributions of *I. cicadae* under three carbon emission scenarios (SSP126, SSP370, and SSP585) for both current conditions and projections for the 2050s and 2070s. Under current climatic conditions, outside of the Xinjiang Uyghur Autonomous Region, Qinghai Province, Ningxia Hui Autonomous Region, Inner Mongolia Autonomous Region, Heilongjiang Province, and Jilin Province, the species’ suitable areas extend across the remaining regions of China. Among these areas, Yunnan, Sichuan, Hunan, Hubei, Guizhou, Jiangxi, Guangxi, Fujian, Anhui, and Zhejiang are identified as having high suitability for *I. cicadae*. The most primary factor influencing the species’ distribution is temperature, followed by precipitation, with key variables including the mean temperature of the driest quarter (bio9), the mean air temperature of the wettest quarter (bio08), and the total precipitation during the wettest season (bio16). This study aims to further elucidate the distribution patterns and environmental influences on *I. cicadae* from a geographical perspective, enhancing its potential as an agricultural crop and providing theoretical insights for future cultivation and conservation strategies.

## Data Availability

The datasets presented in this study can be found in online repositories. The names of the repository/repositories and accession number(s) can be found in the article/supplementary material.

## Footnotes

^1^ https://www.gbif.org/

^2^ http://www.earthol.com/

^3^ http://www.worldclim.org/
